# Editorial: Tumor microenvironment, inflammation, and resistance to immunotherapies

**DOI:** 10.3389/fonc.2023.1215332

**Published:** 2023-05-31

**Authors:** Julie Decock, Giuseppina Comito, Apostolos Zaravinos

**Affiliations:** ^1^ Translational Cancer and Immunity Center (TCIC), Qatar Biomedical Research Institute (QBRI), Hamad Bin Khalifa University (HBKU), Qatar Foundation (QF), Doha, Qatar; ^2^ College of Health and Life Sciences (CHLS), Hamad Bin Khalifa University (HBKU), Qatar Foundation (QF), Doha, Qatar; ^3^ Department of Experimental and Clinical Biomedical Sciences, School of Mathematical, Physical and Natural Sciences, University of Florence, Florence, Italy; ^4^ Department of Life Sciences, School of Sciences, European University Cyprus, Nicosia, Cyprus; ^5^ Cancer Genetics, Genomics and Systems Biology Laboratory, Basic and Translational Cancer Research Center (BTCRC), Nicosia, Cyprus

**Keywords:** tumor microenvironment, inflammation, resistance, immunotherapies, immunosuppression, cancer

The tumor microenvironment (TME) refers to the complex ecosystem surrounding a tumor, including stromal cells, blood vessels, extracellular matrix, and different types of immune cells such as T-cells, B-cells, dendritic cells, neutrophils, natural killer cells, myeloid-derived suppressor cells, and tumor-associated macrophages. Cancer cells exploit the inflammatory mechanisms present in the TME to promote their growth and survival. In turn, immunotherapies, including immune checkpoint inhibition (ICI), adoptive cell transfer (ACT), and genetically-modified T-cell receptor (TCR) and chimeric antigen receptor (CAR-T) based therapies, aim to modulate the immune system to better recognize and eliminate cancer cells. However, the molecular profile of cancer cells affect the TME, hampering the response to these therapies. The causes of immunotherapy resistance remain unclear, but immune dysregulation within the TME, the tumor mutational landscape, inflammation, hypoxia, and epithelial-mesenchymal transition (EMT) have been implicated. Understanding the key immunosuppressive and resistance mechanisms associated with the TME is crucial to develop new therapeutic strategies, limit immune escape, and tailor effective treatments.

This Research Topic aims to provide new insights into the interplay of cancer cells and immune cells within the TME and its impact on resistance to immunotherapeutic approaches.

One of the major mechanisms by which tumor cells can shape the tumor-immune microenvironment in favor of tumor progression is through increased infiltration and polarization of immunosuppressive cells. Here, Cai et al. demonstrated that cancer cell lines release branched-chain α-ketoacids (BCKAs) that affect macrophage polarization in a MCT1-dependent manner whereby α-ketoisocaproate (KIC) and α-keto-β-methylvalerate (KMV) induce pro-tumoral polarization of macrophages whereas α-ketoisovalerate (KIV) exert a pro-inflammatory effect on macrophages, suggesting that cancer-derived BCKAs should be selectively targeted to optimize the anti-tumor immune response. In turn, Qin et al. found that the serine protease PRSS23 is associated with worse prognosis in gastric cancer, supports tumor cell proliferation and invasion, and promotes infiltration of immunosuppressive M2-type macrophages through increased expression and secretion of FGF2. On the other hand, Geng et al. demonstrated how expression of the tumor suppressor RARRES1 could be exploited to enhance the recruitment of anti-tumorigenic type 1 macrophages and reduce the viability of kidney renal clear cell carcinoma cells (KIRC). Although KIRC is the most frequently diagnosed subtype of renal cell carcinoma, the need for diagnostic biomarkers remains unmet and was addressed by Wang et al. who established a 13-gene diagnostic model using cell death-related genes. While NK cells been mostly investigated in relation to their direct anti-tumorigenic functions, Lindsay et al. show that NK cells also play an important role in the maturation of antigen presenting cells during immune responses to early-stage tumors, reducing the development of anergic T cells and improving tumor control and T cell responses.

Given the complexity and dynamic nature of the tumor-immune microenvironment, major efforts are invested towards the identification of biomarkers that can predict the immune composition and contexture of tumors. For instance, Zhong et al. found that elevated expression of FAM110A was associated with the expression of multiple immune checkpoint genes and abundance of tumor-infiltrating immune cells across multiple types of cancer, especially in liver hepatocellular carcinoma. The diverse roles of immune checkpoints in different immune cells were reviewed in more detail by Guo et al. who highlighted the importance of gaining a better understanding of immune checkpoint expression in relation to immune checkpoint blockade. Further, An et al. defined a gene signature score for tertiary lymphoid structures in bladder cancer that correlates with immune cell infiltration, and predicts clinical outcome and response to immunotherapy and chemotherapy. In a second bladder cancer study in this Research Topic, Chang et al. identified a novel immune and inflammatory responses signature (IIRS) that could independently predict overall survival, immunotherapy and chemotherapy response and classify patients with poor clinical and histopathological features. In addition, Georgoulias and Zaravinos examined the expression of various immune receptors, immune-cell fractions, immune-related signatures and mutational signatures across cutaneous melanomas with diverse tumor mutation burdens (TMB) and found that patients with low TMB who are considered to be less responsive to immunotherapy could still benefit from immune-based interventions thanks to pre-existing T-cell immunity. Furthermore, Fang et al. identified a prognostic gene signature associated with iron-dependent regulated cell death, ferroptosis, in triple negative breast cancer which strongly correlated with immunological features and could predict response to anti-cancer treatment. Finally, Kimm et al. observed alterations in the composition of monocyte subpopulations and abundance of monocytic myeloid-derived suppressor cells (mMDSCs) following interstitial brachytherapy or radiofrequency ablation of hepatocellular carcinoma, suggesting that liquid biopsy of monocytes may provide information on the inflammatory response to local ablation.

In addition to modulation of the cellular components of the tumor microenvironment, dysregulation of the extracellular matrix can impact anti-tumor immunity. Here, Donelan et al. discuss how hyaluronan-enriched stroma contributes to tumor growth and progression through the promotion of cancer inflammation, angiogenesis and tumor-associated immune suppression. In this context, Nath et al. demonstrated that inflammation in the bone marrow of N-ethyl-N-nitrosourea-induced leukemic mice could be reduced by treatment with ethanolic olive leaves extract, thereby decreasing the expression of anti-apoptotic proteins.

Overall, the studies in this Research Topic collectively improve our current understanding of the key mechanisms involved in resistance to immunotherapeutic approaches and highlight potential prognostic biomarkers for treatment response. These findings can inform the development of new therapeutic strategies to overcome resistance and improve patient outcomes.

## Author contributions

AZ, JD and GC contributed to writing the manuscript. All the authors proofread the manuscript and approved the submitted version.

